# Mechanical Properties of the Carbonized Layer Formed by Ion Flow Orientated at Different Angles to the Polyurethane Surface

**DOI:** 10.3390/polym16010078

**Published:** 2023-12-26

**Authors:** Vyacheslav S. Chudinov, Igor N. Shardakov, Yaroslav N. Ivanov, Ilya A. Morozov, Anton Y. Belyaev, Irina O. Glot

**Affiliations:** 1 Institute of Continuous Media Mechanics, Ural Branch of Russian Academy of Science, Academician Korolev Street 1, 614013 Perm, Russia; shardakov@icmm.ru (I.N.S.); ilya.morozov@gmail.com (I.A.M.); belyaev@icmm.ru (A.Y.B.); glot@icmm.ru (I.O.G.); 2 Department of Computational Mathematics, Mechanics and Biomechanics, Perm National Research Polytechnic University, Komsomolsky Prospect 29, 614990 Perm, Russia; 3 Faculty of Mechanics and Mathematics, Perm State University, Bukireva Street 15, 614990 Perm, Russia; jaroslawrussia@gmail.com

**Keywords:** coatings, mechanical properties, nanomaterials, polyurethane, ion-plasma treatment

## Abstract

Polymer materials are widely used in medicine due to their mechanical properties and biological inertness. When ion-plasma treatment is used on a polymer material, a carbonization process occurs in the surface nanolayer of the polymer sample. As a result, a surface carbonized nanolayer is formed, which has mechanical properties different from those of the substrate. This layer has good biocompatibility. The formation of a carbonized nanolayer on the surface of polymer implants makes it possible to reduce the body’s reaction to a foreign body. Typically, to study the properties of a carbonized layer, flat polymer samples are used, which are treated with an ion flow perpendicular to the surface. But medical endoprostheses often have a curved surface, so ion-plasma treatment can occur at different angles to the surface. This paper presents the results of a study of the morphological and mechanical properties of a carbonized layer formed on a polyurethane surface. The dependence of these properties on the directional angle of the ion flow and its fluence has been established. To study the surface morphology and elastic properties, methods of atomic force microscopy and methods of elasticity theory were used. The strength properties of the carbonized layer were studied using a stretching device combined with a digital optical microscope.

## 1. Introduction

Polymer materials, such as polyurethanes, polytetrafluoroethylene, polypropylene andpolyetheretherketone, are widely used in medicine due to their mechanical properties and biological inertness [1,2,3,4,5]. For example, polymers are used to make blood vessel prostheses, prosthetic heart valves, meshes for abdominal and inguinal hernia repair complications, breast prostheses, intervertebral cages, etc. [6,7,8,9].

According to the international standard [10], medical polymer materials and products made from them are considered sufficiently biocompatible for use in medical practice, but the risk of the body rejecting such implants remains quite high [11,12]. The reason for these processes is a foreign body reaction which begins with a change in the conformation in the first layer of adsorbed proteins on the surface of the implant and in their desorption and replacement with proteins of the complement system [13].

One solution to the problem of the foreign body reaction is the use of ion-plasma treatment of medical implants, which makes it possible to increase the biocompatibility of the polymer surface. As a result of ion-plasma treatment, carbonization of the surface layer of the polymer occurs and a carbonized hydrophilic nanolayer is formed that is capable of covalently binding adsorbed proteins in the native conformation. This nanolayer has improved biocompatibility compared to the polymer substrate [14,15,16,17].

The mechanical properties of the carbonized nanolayer differ significantly from the properties of the polymer substrate. It is known that the rigidity of a carbonized nanolayer can be several orders of magnitude greater than that of the polymer substrate [18]. Also, ion-plasma treatment of the polymer changes the surface morphology, leading to the formation of folds. The authors of a number of studies [19,20,21,22,23] have made the assumption that this occurs due to the loss of stability of the carbonized nanolayer caused by the action of residual compressive stresses. It should be noted that the quality of interaction of the implant with the living tissue of the body is determined by both the mechanical properties and the morphology of the surface of the carbonized layer.

When forming a carbonized layer on the surface of medical endoprostheses, one has to face another problem. Since these devices have a complex spatial shape formed by curved surfaces, during the plasma treatment of such objects the ion flow falls on different parts of the surface at different angles. It is known that a change in the angle of ion flow can affect the surface morphology [24]. Thus, the properties of such a device after ion-plasma treatment may turn out to be significantly heterogeneous.

This paper presents the results of our study of the rigidity and ultimate deformation properties of polyurethane samples with a carbonized surface layer formed as a result of treatment with a flow of nitrogen ions at different angles. In addition, the surface morphology of the formed carbonized layer is studied.

## 2. Materials and Methods

**Figure 1:**
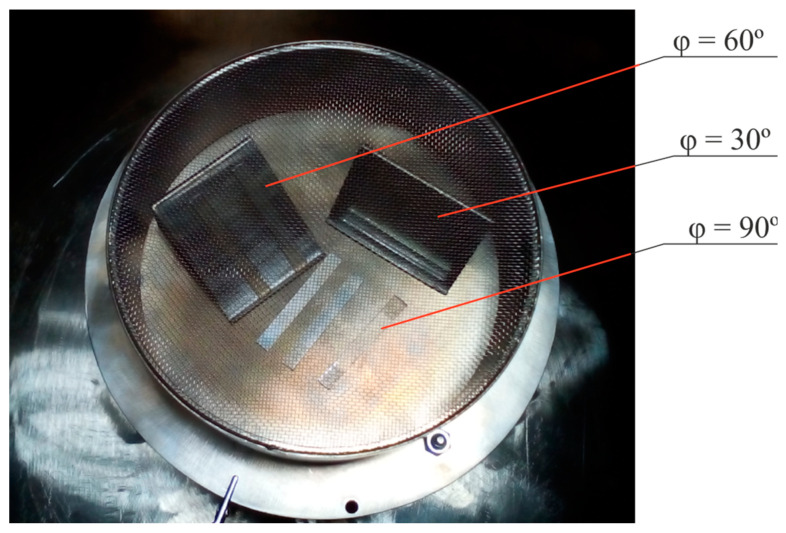
Photo of a vacuum chamber electrode of an ion-plasma installation with polyurethane samples located at different angles φ relative to the ion flow.

Polyurethane samples EP SKU PT-74 (Elastoplast Ltd., Perm, Russia) were synthesized using casting technology [25,26]. They were plates with a thickness of 2 to 3 mm. A carbonized layer was formed on the surface of the samples during their treatment on the ion-plasma installation VSIO-20KV-100NS (Imbiocom Ltd., Perm, Russia). The samples were placed in a vacuum chamber (reactor) on special supports, the surface of which had a different tilt angle φ relative to the direction of ion flow (Figure 1). This ensured the impact of the ion flow at different angles to the surfaces of the samples. The angles were 30°, 60° and 90°.

To carry out ion-plasma treatment, air was pumped out of the reactor until the pressure in the chamber reached 10^−3^ Pa. After this, nitrogen was supplied into the reactor through a needle leak until the pressure in the chamber became 5 × 10^−1^ Pa. To ionize nitrogen and initiate the directional movement of the ion flow, rectangular negative high-voltage pulses with a frequency of 100 Hz were applied to the electrode. The pulse duration was 20 μs and the amplitude was 20 kV. Next, the ions were accelerated through a metal mesh, under which the samples were placed on inclined supports. The fluence (the total number of ions introduced per unit surface of the sample during the entire processing time) varied from 5 × 10^14^ ions/cm^2^ to 10^16^ ions/cm^2^ and the energy of the ion flux was 20 keV.

**Figure 2:**
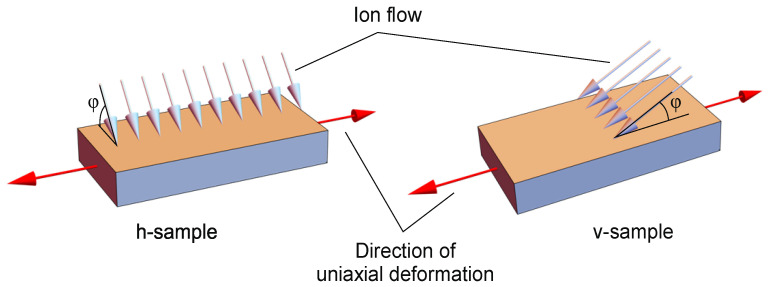
Two types of samples (h-sample and v-sample) with different orientations of the ion flow relative to the direction of uniaxial deformation: blue arrows—direction of ion flow, red arrows—direction of uniaxial tension.

To assess the anisotropy of the ultimate deformations of the surface layer, rectangular polyurethane samples had different locations on supports. For samples named v-samples, the direction of the projection of the ion flow onto the sample plane coincides with the direction of uniaxial deformation. Accordingly, for samples named h-samples, the projection of the ion flow onto the sample plane is oriented perpendicular to the direction of uniaxial deformation (Figure 2).

**Figure 3:**
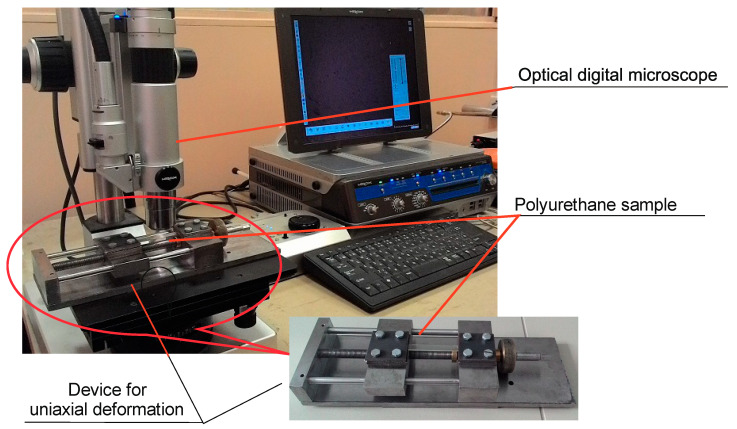
Optical digital microscope with a device for uniaxial tension.

A special device was developed and created for uniaxial tension of samples, which makes it possible to set the required level of sample deformation. The device was placed in the groove of the stage of a Hirox digital optical microscope (Hirox Co., Ltd., Tokyo, Japan), which made it possible to observe the appearance of changes in the surface layer directly during the deformation process and to obtain photographs at a given level of deformation (Figure 3).

To study the surface topography of plasma-treated and untreated samples, Ntegra Prima atomic force microscope (NT-MDT Ltd., Zelenograd, Russia) was used in semi-contact operating mode. In our study, the size of the observed area on the surface of the samples was 20 × 20 μm (with a resolution of 500 × 500 pixels). Plasma-treated samples were studied using FMG01 probes (NT-MDT Ltd., Zelenograd, Russia) with tip radius *ρ* = 10–12 nm and cantilever stiffness k = 3.5 N/m. When working with untreated samples, softer ScanAsyst-Air probes (Bruker, Billerica, MA, USA) were used. Their characteristics were as follows: *ρ* = 4 nm and k = 0.4 N/m.

The mechanical properties of the surfaces of treated and untreated samples were studied using an atomic force microscope (AFM) in indentation mode. To accomplish this, an area of 3 × 3 μm (with a resolution of 300 × 300 pixels) without sharp changes in height was selected on the surface of the sample. In this mode, the probe pressed on the surface at a given frequency. For untreated samples, the indentation force was 0.4 nN, and for modified ones, the indentation force was ~3–5 nN. This mode of using AFM makes it possible to obtain experimental information about the correspondence between the vertical displacement *U* of the cantilever (normal to the sample surface) and the specified value of the pressing force *F*. This information was the basis for determining the elastic modulus *E_c_* of the created carbonized layer. The elastic modulus was determined by using two methods.

First method. This method is based on the analytical solution of the Hertz contact problem—the indentation of an elastic spherical body (cantilever tip) into an elastic half-space (carbonized layer) [27]. From this solution follows an expression for determining the elastic modulus of the carbonized layer *E_c_*:

(1) $$E_{c}=\frac{F\cdot E_{s}(1-\nu_{c}^{2})}{\left(4/3\cdot E_{s}\cdot \rho^{\frac{1}{2}}\cdot U^{\frac{3}{2}}\right)-F(1-\nu_{s}^{2})}$$

where *F* is the force acting on the cantilever tip when pressed into the surface of the sample; *U* is the displacement of the cantilever tip normal to the surface of the sample due to the action of force *F*; *ν_s_* and *ν_c_* are Poisson’s ratios, respectively, for the cantilever tip material and the carbonized layer; *E_s_* is the elastic modulus for the cantilever tip material; *ρ* is the radius of the spherical tip of the cantilever. The values of the physical and mechanical parameters required to calculate the right side of Formula (1) are given in Table 1.

The validity of the possibility of using relation (1) to determine the elastic modulus of a carbonized layer is based on the fact that the cantilever tip radius is, as a rule, an order of magnitude smaller than the thickness of this layer. It should be noted that the same relationship (1), and with more justification, was used to determine the elastic modulus of polyurethane *E_p_* in the case of untreated samples.

**Figure 4:**
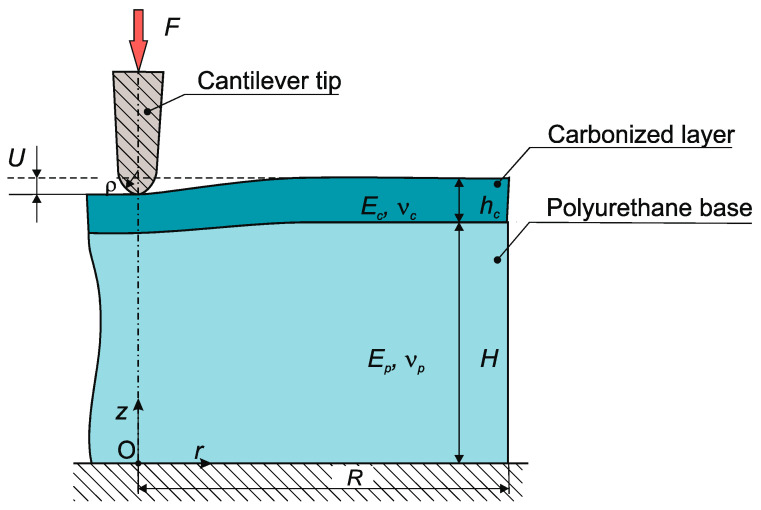
Calculation scheme for solving the axisymmetric contact problem of indenting a cantilever tip into the surface of a polyurethane sample in a cylindrical coordinate system (*r*,*O*,*z*).

Second method. This method is based on numerical solutions of the contact problem of pressing a probe into the surface of a polyurethane sample (treated or not treated with a plasma flow) with a given vertical force *F*. The structural diagram of the problem being solved is presented in Figure 4. The solution of the problem is carried out within the framework of the isotropic theory of elasticity, taking into account axial symmetry in a cylindrical coordinate system (*r*,*O*,*z*). The initial data for the problem are the geometric parameters of the interacting elements (the thickness of the carbonized layer *h_c_* and the polyurethane substrate *H*; the outer radius of the axisymmetric computational region *R*; the radius of the spherical tip of the cantilever *ρ*) and the physical and mechanical characteristics of the materials (Poisson’s ratios, *ν_s_*, *ν_c_*, *ν_p_*, and elasticity moduli, *E_s_*, *E_c_*, *E_p_*_,_ for the cantilever tip, carbonized layer and polyurethane substrate, respectively). The physical and mechanical properties of the materials of the interacting elements are given in Table 1. The radius and thickness of the calculation region are *R* = 5000 nm and *H* = 2000 nm. They were determined on the basis of a numerical experiment so that the relationship between the indentation force and displacement did not depend on the boundary conditions of the computational domain. The radius of curvature of the cantilever tip was *ρ* = 10–12 nm. The thickness of the carbonized layer *h_c_* was set based on the results of mathematical modeling of the depth of the penetration of the ions into the molecular structure of the polymer substrate, as described below.

The boundary conditions for the axisymmetric region under consideration are as follows. The lower boundary at *z* = 0 is rigidly fixed; on a cylindrical surface at *r* = *R* there are no external forces and kinematic links. On the contact surface of the carbonized layer and the polyurethane substrate at *z* = *H*, the conditions of continuity of the displacement vector and contact interaction forces are specified. At *z = H + h_c_*, the conditions for the contact deformation interaction of the cantilever tip with the surface of the sample are specified, the area of which is determined iteratively in the process of solving the problem. The remaining surface at *z = H + h_c_* is free from external forces and kinematic links. A vertical distributed load is specified on the upper end of the cantilever tip; its integral value is equal to *F*. The rest of the tip surface, with the exception of the local area of contact with the surface layer of the sample, is free from loads and kinematic links. The magnitude of the vertical load *F* is set by the AFM readings when the cantilever tip is pressed into the surface layer.

The numerical implementation of this problem of the contact deformation interaction of the cantilever tip with the surface of the sample was carried out by the finite element method using the Ansys software package (version 2022 R2).

To determine the elastic modulus of the carbonized layer, the following algorithm was developed. A set of numerical solutions to the problem of pressing a cantilever tip into a sample with a carbonized layer was obtained for different values of the elastic modulus of the layer. These values were taken from an interval known to contain the true value corresponding to a specific layer on a specific sample. For each value of the modulus, a pair of quantities was determined—the vertical force *F* acting on the cantilever tip and the vertical displacement *U*. The desired value of the modulus *E_c_* was taken to be the value from the used set that gives the values of the pair of *F* and *U* closest to the experimentally measured values with an AFM. The presented fundamental structure of the algorithm assumes the possibility of using various options for accelerating the solution and increasing the accuracy of modulus value calculations.

**Figure 5:**
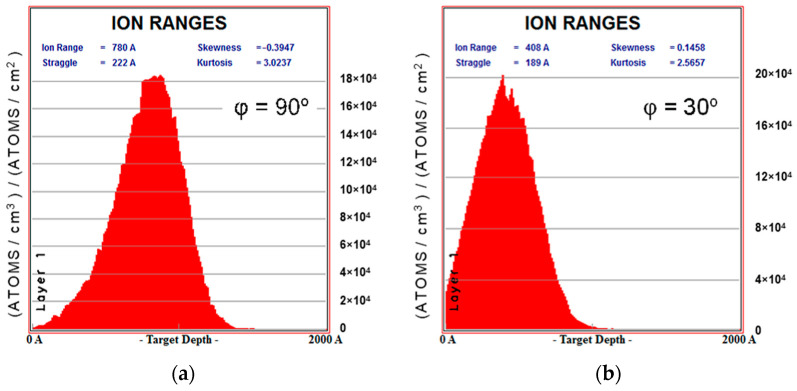
Distribution density of the penetration depth of nitrogen ions into the surface layer of a polyurethane sample: (**a**) ion flow directed at an angle of 90°; (**b**) ion flow directed at an angle of 30°.

Method for determining the thickness of the carbonized layer. When numerically modeling the response of a sample to the influence of an indenter, it is important to correctly set the thickness of the modified layer. This value was estimated using the TRIM software tool (version SRIM-2013) [28], which allows one to calculate the trajectories of ion penetration into the substrate. For a flow of nitrogen ions with an energy of 20 keV, histograms of the distribution of the penetration depth of ions into the polyurethane layer were obtained. These distributions were calculated for three values of the flow direction angle to the sample surface: *φ* = {30°, 60°,90°}. Figure 5 shows these graphs for the angles of 90° and 30°.

**Figure 6:**
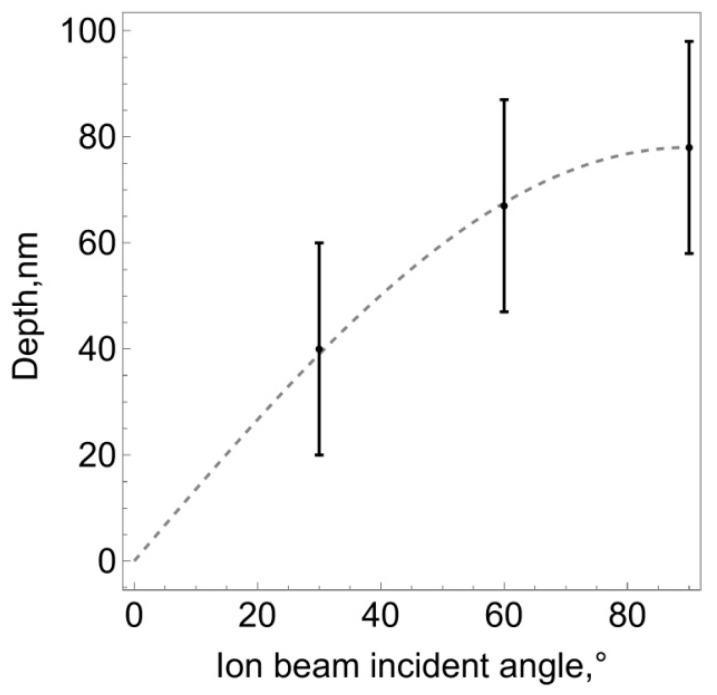
Ion penetration depth depending on the inclination of the ion flow relative to the sample surface. Dots and vertical bars represent average values and standard deviations. The dashed line is dependence $h_{c}\prime =h_{c}\sin\varphi$.

Statistical analysis of the obtained distributions made it possible to estimate the average values of ion penetration depth, which can be considered to bethe theoretical value of the thickness of the modified layer *h_c_*. For the three indicated angles, this value was 40, 68 and 78 nm, respectively. Figure 6 shows the dependence of the thickness of the modified layer on the angle of ion flow.

The obtained result is quite consistent with geometric considerations. An ion flow directed normal to the surface ensures penetration into the substrate to an average depth *h_c_*. If the flow bombards the surface at an angle φ≠90°, the ions still travel a distance *h_c_* in the substrate, but the thickness of the layer normal to the surface will be $h_{c}\prime =h_{c}\sin\varphi$. The graph shown in Figure 6 with a dashed line demonstrates exactly this dependence.

**Figure 7:**
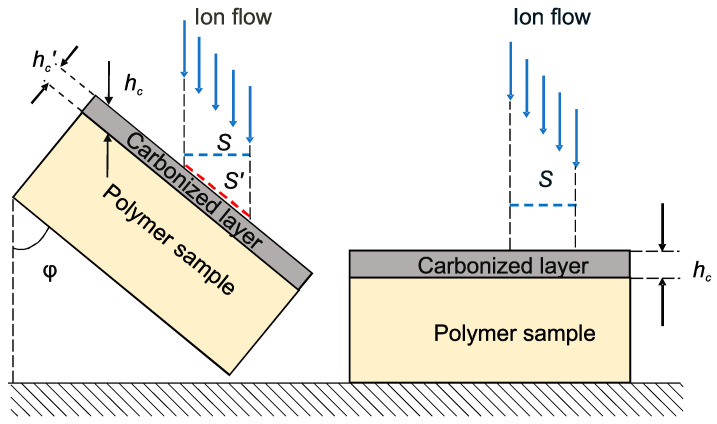
Schematic representation of ion-plasma treatment at different orientations of the ion flow relative to the surface of the material.

From the geometric considerations shown in Figure 7, another important conclusion follows. If an ion flow with density *i* is directed perpendicular to the surface, then a number of ions $I=iSh_{c}$ enters the surface layer with area *S* and depth *h_c_*. An ion flow of the same density, directed at a different angle φ≠90°, is distributed over the surface S′=S/sin⁡φ and penetrates to the depth $h_{c}\prime =h_{c}\sin\varphi$. In this case, the number of ions penetrating into the substrate within this area will be the same: $I\prime =iSh_{c}=I$. But since the degree of carbonization of the treated layer depends on the total number of ions penetrated, it can be expected that when the direction of the ion flow changes, only the thickness of the modified layer should change, but its mechanical properties should remain the same.

## 3. Results and Discussion

**Figure 8:**
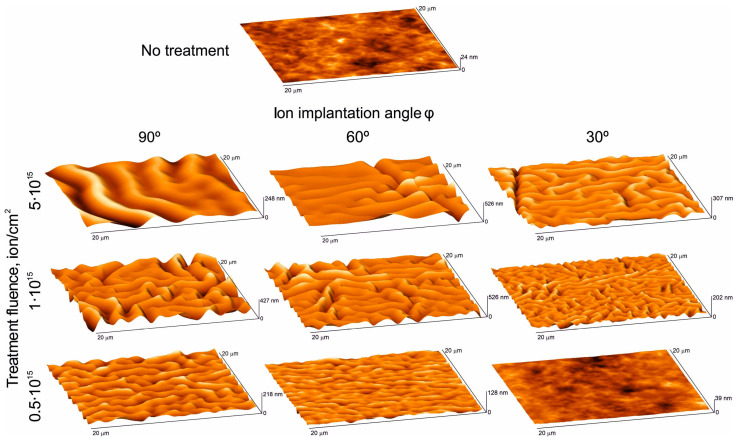
3D-AFM image of a 20 × 20 µm fragment of the surface of samples treated with an ion flow with different values of and flow angles.

**Figure 9:**
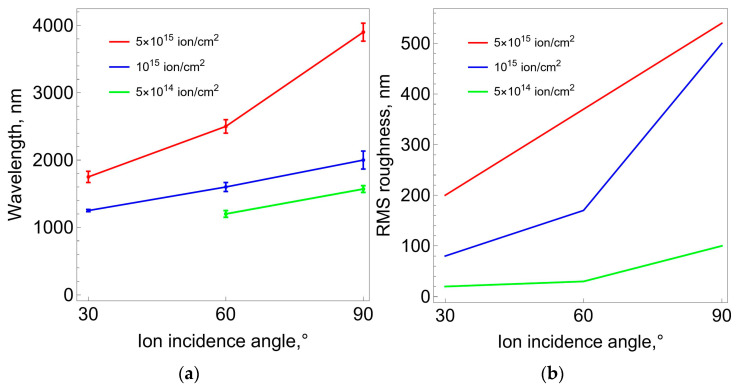
Morphological characteristics of polyurethane samples depending on the ion flow angle and treatment fluence: (**a**) wavelength of folds (average values and standard deviations); (**b**) surface roughness (RMS value).

The study of the surface relief features of polyurethane samples (modified and unmodified) was carried out using an atomic force microscope in accordance with the description in Section 2. Figure 8 shows high-resolution tone photographs of the samples’ surface after treatment with nitrogen ions of varying intensities (fluence) at different flow angles to the surface. These photos clearly demonstrate the significant influence of fluence level and angle on surface morphology. It can be stated that after treating the samples with a plasma flow, irregularly arranged folds appear on their surface. At the same time, it is clearly seen that with each processing mode, a dominant type of fold with certain characteristic geometric dimensions forms. The general trend is an increase in these characteristic sizes with increasing fluence. This trend is confirmed by the results of photo processing, which is presented in the form of graphs in Figure 9.

The characteristic wavelength of the folds was estimated from the obtained micrographs by measuring the distance between adjacent wave crests. For each sample, 30 measurements were made, from which the average wavelength and standard deviation were estimated. Surface roughness was assessed in a similar manner. Using microphotographs of the samples, the difference in height between the wave crest and trough was measured and compared with the average height value. Measurements were performed at 30 points on the sample surface. The root-mean-square roughness value (RMS-roughness) was calculated using the following formula:

$$RMS=\sqrt{1/N\cdot \sum_{i=1}^{N}z_{i}^{2}}$$

where *z_i_* is the deviation of the height of the peak or the depth of the valley from the profile center line and where *N* is the number of measurements.

**Figure 10:**
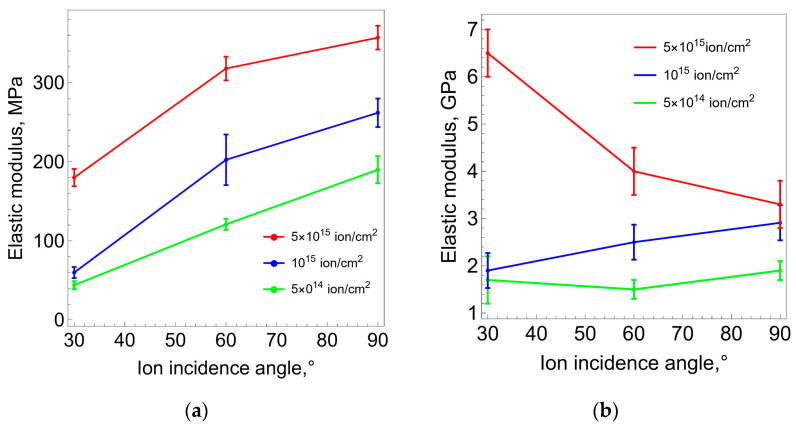
Modulus of elasticity of the carbonized layer (average values and standard deviations) depending on the angle of ion flow and processing fluence: (**a**) solution to the Hertz problem; (**b**) calculation using the FEM model.

Figure 10 shows the values of the elastic modulus of the carbonized layer depending on the fluence of ion-plasma treatment and the direction angle of the ion flow. The dependences were obtained from AFM results, which were processed by using two methods presented in Section 2. The results in Figure 10a were obtained via the first method, based on the analytical solution of the Hertz problem on the contact interaction of an elastic sphere with an elastic half-space, which are a cantilever tip and a carbonized layer. The results in Figure 10b were obtained via the second method, which used numerical finite element solutions to the problem of contact interaction between the cantilever tip and the surface of the processed sample. In this approach, unlike the first method, the real values of the thicknesses of the polyurethane substrate and the carbonized layer are taken into account. From a comparison of the obtained dependencies it follows that both the first and the second methods show that the value of the elastic modulus of the carbonized layer increases with increasing fluence. But the quantitative values obtained based on numerical solutions are almost an order of magnitude larger. When obtaining an analytical solution, it is assumed that the cantilever tip is embedded in a homogeneous medium, the properties of which correspond to the carbonized layer. The numerical solution considers precisely the two-layer structure of the sample, in which the rigidity of the carbonized layer is significantly greater than the rigidity of the substrate. Thus, we believe that taking into account the two-layer structure of the sample significantly refines the values of the desired elastic modulus of the carbonized layer.

**Figure 11:**
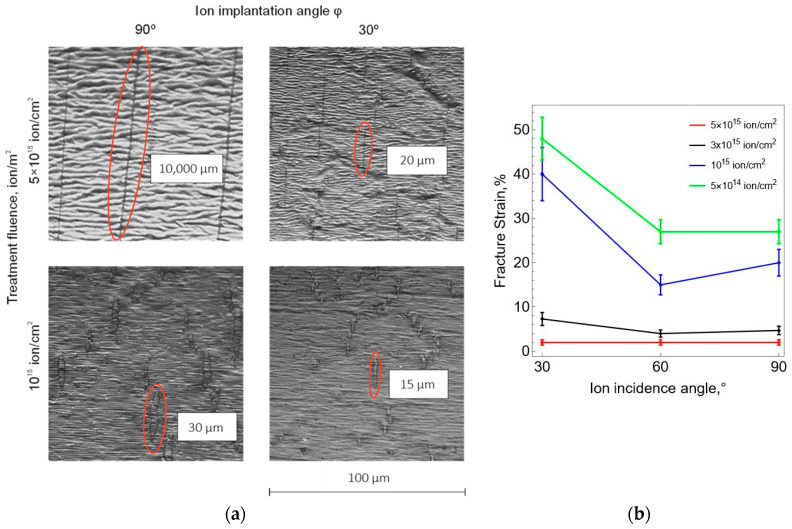
The surface of the carbonized layer at different fluences of ion-plasma treatment and the inclination of the ion flow: (**a**) micrographs of the surface with primary cracks; (**b**) ultimate strain (average value and standard deviation).

A series of experiments was carried out to determine the ultimate deformations of the carbonized layer on the surface of the polyurethane samples. The experiments were carried out using a digital optical microscope combined with a miniature stretching device according to the algorithm presented in Section 2. Figure 11a shows micrographs of the surface of the carbonized layer on deformed samples at the moment of the formation of the first cracks. All cracks are located perpendicular to the direction of tension, which means that they are normal rupture cracks. From a comparison of the results obtained, it follows that the length of cracks increases with an increase in the fluence of ion-plasma treatment and decreases with a decrease in the direction angle of the ion flow. The results in Figure 11b demonstrate patterns of change in the ultimate strain of the carbonized layer, which is the strain corresponding to the appearance of the first cracks on the surface of the sample. The ultimate strain decreases as the flow direction angle tends to 90° (perpendicular to the surface). In this case, an increase in fluence also leads to a decrease in the ultimate strain.

**Figure 12:**
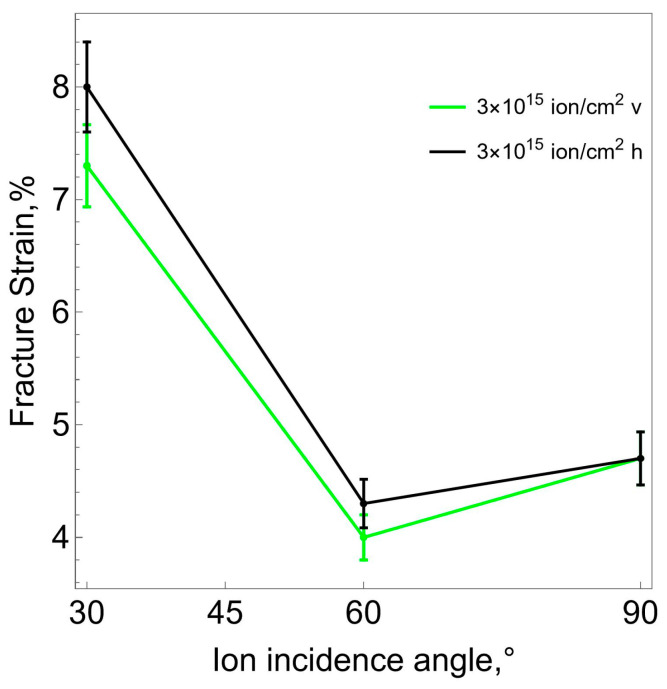
Ultimate strain of the surface layer depending on the direction of the ion beam.

Our experiments have shown that the surface layer formed during ion-plasma treatment has a certain anisotropy. The strains corresponding to the onset of surface crack formation are lower for the samples stretched in the direction coinciding with the direction of the ion flow (v-samples). The samples stretched in a direction perpendicular to the ion flow (*h*-samples) begin to crack at greater deformations. This dependence is demonstrated in Figure 12.

## 4. Conclusions

Based on the results of the numerical simulation, the distribution of the density of nitrogen ions over the depth of penetration into the surface of polyurethane samples during ion-plasma treatment was determined. The average value of the ion penetration depth was taken as the thickness of the carbonized layer. The dependence of the thickness of the carbonized layer on the angle of inclination of the ion flow to the sample surface has been established.

Based on the AFM results, it was established that after treating polyurethane samples with a plasma flow, a carbonized layer with unevenly spaced folds is formed. The dependences of the characteristic dimensions of the folds (length and depth) on the fluence and the directional angle of the ion flow were obtained. The general trend is an increase in these characteristics with increasing fluence and angle of the ion flow. It should be noted that there is still no clear understanding of the mechanisms of the formation of these folds.

Processing and analysis of the AFM results by using two methods (Hertz model and finite element analysis) made it possible to estimate the elastic modulus of the carbonized layer depending on the fluence and the direction angle of the ion flow. From a comparison of the obtained dependencies it follows that, in both cases, the elastic modulus of the carbonized layer increases with increasing fluence. But the quantitative module obtained from numerical solutions is almost an order of magnitude greater. It follows from this that taking into account the real thicknesses of the carbonized layer and the polyurethane substrate significantly refines the values of the desired elastic modulus of the carbonized layer.

An analysis was made of the results of the uniaxial deformation of the modified polyurethane samples using a stretching device combined with a digital optical microscope. The moment of appearance of the first cracks in the carbonized layer was recorded. All cracks were located perpendicular to the stretch direction, i.e., normal rupture cracks are realized. The length of cracks increases with increasing fluence and decreases with decreasing angle of ion flow direction. The ultimate strain, characterized by the appearance of the first cracks in the carbonized layer, have been determined. A pattern is shown of a decrease in the ultimate strain as the flow direction tends to 90° (perpendicular to the surface). An increase in fluence also leads to a decrease in the ultimate strain.

Uniaxial tensile experiments showed that the surface layer formed during ion- plasma treatment has a certain anisotropy. The limiting strains corresponding to the onset of surface crack formation are lower for samples stretched in the direction coinciding with the direction of ion flow (v-samples). Specimens stretched in a perpendicular direction begin to crack at larger strains. However, it should be noted that analysis of the possibility of anisotropy manifestations in the carbonized layer requires a more detailed (large-scale) study.

## Figures and Tables

**Table 1:** Physical and mechanical properties of the materials of interacting elements.

Element	Poisson’s Ratio	Modulus of Elasticity, GPa
Cantilever tip	*ν_s_* = 0.26	*E_s_* = 110
Carbonized layer	*ν_c_* = 0.31	*E_c_* ∈ (0.1; 5)
Polyurethane base	*ν_p_* = 0.45	*E_p_* = 0.025

## Data Availability

The data presented in this study are available on request from the corresponding author.

## References

[1] Shekhawat D., Singh A., Bhardwaj A., Patnaik A. (2021). A Short Review on Polymer, Metal and Ceramic Based Implant Materials. IOP Conf. Ser. Mater. Sci. Eng..

[2] Sarem M., Moztarzadeh F., Mozafari M. (2013). How can genipin assist gelatin/carbohydrate chitosan scaffolds to act as replacements of load-bearing soft tissues. Carbohydr. Polym..

[3] Yazdanpanah A., Amoabediny G., Shariatpanahi P., Nourmohammadi J. (2014). Synthesis and Characterization of Polylactic Acid Tubular Scaffolds with Improved Mechanical Properties for Vascular Tissue Engineering. Trends Biomater. Artif. Organs..

[4] Zarrintaj P., Urbanska A.M., Seyed S., Goodarzi V., Reza M., Mozafari M. (2018). A facile route to the synthesis of anilinic electroactive colloidal hydrogels for neural tissue engineering applications. J. Colloid Interface Sci..

[5] Gholipourmalekabadi M., Samadikuchaksaraei A., Seifalian A.M., Urbanska A.M., Ghanbarian H. (2017). Silk fibroin/amniotic membrane 3D bi-layered artificial skin. Biomed. Mater..

[6] Kakisis J.D., Antonopoulos C., Mantas G., Alexiou E., Katseni K., Sfyroeras G., Moulakakis K., Geroulakos G. (2017). Safety and efficacy of polyurethane vascular grafts for early hemodialysis access. J. Vasc. Surg..

[7] Kütting M., Roggenkamp J., Urban U., Schmitz-Rode T., Steinseifer U. (2011). Polyurethane heart valves: Past, present and future. Expert Rev. Med. Devices.

[8] Rezvova M.A., Ovcharenko E.A. (2018). Polimernye protezy klapanov serdca: Sostoyanie i perspektivy [Polymer heart valve prostheses: Status and prospects]. Vestn. Transplantologii I Iskusstv. Organov.

[9] Antonova L.V., Nasonova M.V., Kudryavceva Y.A., Golovkin A.S. (2012). Vozmozhnosti ispol’zovaniya polioksialkanoatov i polikaprolaktona v kachestve sopolimernoj osnovy dlya sozdaniya tkaneinzhenernyh konstrukcij v serdechno-sosudistoj hirurgii [Possibilities of using polyhydroxyalkanoates and polycaprolactone as a copolymer base for creating tissue engineering structures in cardiovascular surgery]. Byulleten’ Sib. Med..

[10] (2018). Biological Evaluation of Medical Devices. Part 1: Evaluation and Testing within A Risk Management Process.

[11] Yoshida S., Hagiwara K., Hasebe T., Hotta A. (2013). Surface modification of polymers by plasma treatments for the enhancement of biocompatibility and controlled drug release. Surf. Coat. Technol..

[12] Jurak M., Wiącek A.E., Ładniak A., Przykaza K., Szafran K. (2021). What affects the biocompatibility of polymers. Adv. Colloid Interface Sci..

[13] Sevastyanov V.I., Kirpichnikov M.P. (2011). Biosovmestimye Materialy [Biocompatible Materials].

[14] Kondyurin A.V., Bilek M. (2015). Ion Beam Treatment of Polymers Application Aspects from Medicine to Space.

[15] Chudinov V.S., Kondyurina I.V., Shardakov I.N., Svistkov A.L., Osorgina I.V., Kondyurin A.V. (2018). Polyurethane Modified with Plasma-Ion Implantation for Medical Applications. Biophysics.

[16] Chudinov V., Kondyurina I., Terpugov V., Kondyurin A. (2019). Weakened foreign body response to medical polyureaurethane treated by plasma immersion ion implantation. Nucl. Instrum. Methods Phys. Res. Sect. B.

[17] Chudinov V.S., Shardakov I.N., Svistkov A.L., Kondyurin A.V. Polyurethane modified by plasma ion implantation. Proceedings of the NANOCON 2018-Conference Proceedings, 10th Anniversary International Conference on Nanomaterials-Research and Application.

[18] Shardakov I., Ivanov Y., Chudinov V., Glot I., Timkina T. (2022). Mechanical characteristics of the carbonized layer on the surface of polyethylene treated by the ion-plasma method. Procedia Struct. Integr..

[19] Gonzalez Henriquez C., Rodriguez-Hernandez J. (2019). Wrinkled Polymer Surfaces Strategies, Methods and Applications: Strategies, Methods and Applications.

[20] González-Henríquez C.M., Rodríguez-Umanzor F.E., Alegría-Gómez M.N., Terraza-Inostroza C.A., Martínez-Campos E., Cue-López R., Sarabia-Vallejos M.A., García-Herrera C., Rodríguez-Hernández J. (2021). Wrinkling on Stimuli-Responsive Functional Polymer Surfaces as a Promising Strategy for the Preparation of Effective Antibacterial. Antibiofouling Surfaces. Polymers.

[21] Moon M.W., Lee S.H., Sun J.Y., Oh K.H., Vaziri A., Hutchinson J.W. (2007). Controlled formation of nanoscale wrinkling patterns on polymers using focused ion beam. Scr. Mater..

[22] Morozov I.A., Mamaev A.S., Bannikov M.V., Beliaev A.Y., Osorgina I.V. (2018). The Fracture of Plasma-Treated Polyurethane Surface under Fatigue Loading. Coatings.

[23] Kondyurin A.V., Maitz M.F., Romanova V.A., Begishev V.P., Kondyurina I.V., Guenzel R. (2004). Drug release from polyureaurethane coating modified by plasma immersion ion implantation. J. Biomater. Sci. Polym. Ed..

[24] Jeong H.C., Park H.G., Jung Y.H., Lee J.H., Oh B.Y., Seo D.S. (2016). Tailoring the Orientation and Periodicity of Wrinkles Using Ion-Beam Bombardment. Langmuir.

[25] Kislicyn V.D., SHadrin V.V., Osorgina I.V., Svistkov A.L. (2020). Analiz mekhanicheskih svojstv poliuretanovyh materialov, izgotovlennyh po rastvornoj i lit’evoj tekhnologiyam [Analysis of the mechanical properties of polyurethane materials manufactured using mortar and injection technologies]. Vestn. Permsk. Univ. Fiz..

[26] Chudinov V.S., Shardakov I.N., Ivanov Y.N., Morozov I.A., Belyaev A.Y. (2023). Elastic Modulus of a Carbonized Layer on Polyurethane Treated by Ion-Plasma. Polymers.

[27] Timoshenko S.P., Goodier J.N. (1970). Theory of Elasticity.

[28] Ziegler J.F., Ziegler M.D., Biersack J.P. (2010). SRIM—The stopping and range of ions in matter. Nucl. Instrum. Methods Phys. Res. Sect. B Beam Interact. Mater. At..

